# ^86^Kr excess and other noble gases identify a billion-year-old radiogenically-enriched groundwater system

**DOI:** 10.1038/s41467-022-31412-2

**Published:** 2022-06-30

**Authors:** O. Warr, C. J. Ballentine, T. C. Onstott, D. M. Nisson, T. L. Kieft, D. J. Hillegonds, B. Sherwood Lollar

**Affiliations:** 1 Department of Earth Sciences, University of Toronto, Toronto, ON M5S 3B1 Canada; 2 Department of Earth Sciences, University of Oxford, Oxford, OX1 3AN UK; 3 Department of Geosciences, Princeton University, Princeton, NJ 08544 USA; 4 Biology Department, New Mexico Institute of Mining and Technology, Socorro, NM 87801 USA; 5 IPGP, Sorbonne Paris Cité, 1 rue Jussieu, 75005 Paris, France

**Keywords:** Biogeochemistry, Geochemistry, Hydrology, Element cycles, Hydrology

## Abstract

Deep within the Precambrian basement rocks of the Earth, groundwaters can sustain subsurface microbial communities, and are targets of investigation both for geologic storage of carbon and/or nuclear waste, and for new reservoirs of rapidly depleting resources of helium. Noble gas-derived residence times have revealed deep hydrological settings where groundwaters are preserved on millions to billion-year timescales. Here we report groundwaters enriched in the highest concentrations of radiogenic products yet discovered in fluids, with an associated ^86^Kr excess in the free fluid, and residence times >1 billion years. This brine, from a South African gold mine 3 km below surface, demonstrates that ancient groundwaters preserved in the deep continental crust on billion-year geologic timescales may be more widespread than previously understood. The findings have implications beyond Earth, where on rocky planets such as Mars, subsurface water may persist on long timescales despite surface conditions that no longer provide a habitable zone.

## Abstract (web summary)

Noble gases confirm billion-year groundwater residence times and external fluxes in deep crustal settings globally with implications for subsurface habitability and economic reservoir formation over planetary timescales both on Earth and beyond

## Introduction

The Precambrian crystalline basement represents approximately 72% of the continental crust by surface area and is estimated to host up to 30% of the total groundwater inventory of the Earth. Annual H_2_ production in these environments via water-rock reactions has recently been shown to be comparable to that of the oceanic crust^1–4^. In these deep crustal environments, long-term radiogenic and other processes of water-rock reactions including serpentinization produce complex fluids characterized by Ca-Na-Cl salinity and products of abiotic organic synthesis such as acetate and formate as well as CH_4_ and higher hydrocarbons via Fischer-Tropsch-type reactions^5–11^. These processes are capable of sustaining chemolithotrophic deep subsurface microbial communities^12,13^. Typically disconnected from the modern surface hydrologic cycle, these fracture fluids accumulate radiogenic noble gases produced as by-products of radioelement (U, Th, K) decay over geologic time, with mean fluid residence times ranging over ka-Ga timescales^2,3,9,14–18^ and have distinctive isotopic and elemental fluid compositions and high concentrations of reduced gases (e.g. H_2_, CH_4_) from long-term, low temperature water-rock interaction^5–9,11,19,20^. Subsurface microbial communities have been identified in these fracture networks and investigations of their genomic lineage is providing insight into the evolutionary history of the deep biosphere and the transport, and timing of isolation of deep life within the planetary crust^20–24^. Due to the long timescales involved, conventional short-lived tracers such as ^3^H, or ^14^C cannot be applied, and instead geochronology of the fluids depends on measuring and interpreting the accumulation of radiogenic ^4^He, ^21^Ne, ^40^Ar, and ^136^Xe, produced through the decay of U, Th, and K in the host rocks^2,14–18^. At Kidd Creek Observatory on the Canadian Precambrian Shield, concordant noble gas-derived residence time estimates from four independent noble gas tracers identified fluids with residence times of up to 1.7 Ga, the oldest free-flowing fluids on record^2,14^. The concordance of the noble gas systems, and other multiple lines of evidence (e.g. δ^18^O, δ^2^H, sulfur isotopes)^2,19^, demonstrated the Kidd Creek fracture network was an example of extended hydrogeologic isolation of fluids. On the other end of the open-closed spectrum, some deep crustal environments are impacted by the penetration of surface-derived paleometeoric water with residence times in the ka-Ma range to depths of more than 1 km^16,17^.

Moab Khotsong Mine (26.9792°S, 26.7815°E) is a gold and uranium mine located in the Witwatersrand Basin, within the Kaapvaal Craton, South Africa. Geologically, the craton formed approximately 3.7–2.6 Ga and consists of granites-greenstones, tonalite-trondhjemite-granodiorite gneisses, mafic-ultramafic rocks and sedimentary rocks^17,25–27^. The Witwatersrand Basin formed due to tectono-magmatic activity between 3.074 and 2.642 Ga^27,28^. The Witwatersrand Supergroup is subdivided into the Central Rand Group, consisting primarily of terrigenous quartzites with minor conglomerates and shales, and overlies the West Rand Group, which consists of transitional marine-to-continental shales and quartzites^26^. Deposition of Witwatersrand Supergroup terminated at 2.714 Ga when the overlying Ventersdorp Supergroup lavas were deposited^28^. The principal economic target at Moab Khotsong is the U-Au-rich Vaal Reef conglomerate, which is stratigraphically located in the middle of the Central Rand Group^26,29^.

Here we report noble gas concentrations and isotopic data from a borehole drilled at the mine into the uppermost West Rand Group at an average inclination of 47° starting at 2.896 km below land surface (kmbls), and extending 810 m to a depth of 3.491 kmbls. This borehole intersects fractures including a major mineralized fracture zone at a depth of 3.213 kmbls in a sub-horizontal mafic intrusion located 50 m above the 2.914 ± 8 Ga Crown Lava^28^. The fracture system releases a Ca-Na-Cl brine (in situ pressure = 102 bars and temperature = 54 °C) with high concentrations of reduced gases such as H_2_, CH_4_, C_2_H_8_. δ^18^O and δ^2^H isotope values for the fluids plot well above the Global Meteoric Water Line (GMWL), confirming that this fracture fluid system is isolated from the surface hydrologic cycle^9^ (Supplementary Tables 1, 4). The noble gas data presented here reveal these fluids have a residence time of >1 billion years, confirm the unambiguous presence of a preserved ^86^Kr excess observed in a free fluid, and reveal that preservation of fluids on billion-year geologic timescales are a feature of the deep continental crust on a global scale. Distinctive patterns in the light versus heavy noble gas signatures here quantify processes controlling hydrogeologic isolation in these deep fracture systems. This is critical to advancing our understanding of the timing and degree to which deep subsurface groundwater systems are open versus closed to the surface hydrosphere, and provide insight into the processes controlling accumulation versus diffusive loss.

## Results

**Fig. 1:**
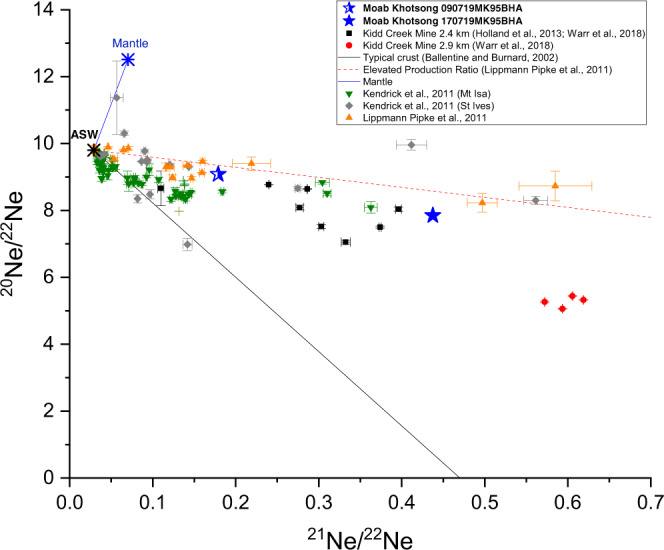
Neon isotope data. Neon signatures confirm that fluids sampled at Moab Khotsong (blue stars) are the result of radiogenic (nucleogenic) production in the crust added to an initial, Air-Saturated Water (ASW) component recharged from the surface, but no discernible mantle contribution. These data are consistent with the elevated crustal ^21^Ne production observed to date only in samples collected from Precambrian cratons, e.g. Kidd Creek^2,14^ (Canadian Shield), fracture fluids from Driefontein, Evander, Kloof, and Mponeng mines in the Witwatersrand Basin^17^ (demonstrated here by the red triangles and dashed red line reproduced from Lippmann-Pipke et al.^18^), and fluid inclusions from both the St. Ives Goldfield in the Yilgarn Craton^33^ (grey diamonds), and the Mt. Isa inlier (green triangles)^34^. Prior to the discovery of these distinctive radiogenic ^21^Ne signatures, radiogenic neon addition to ASW in average continental crust globally fell along the lower trend represented here by the solid black line. Published data from Kidd Creek fracture fluids^2,14^ are plotted for context (black squares and red circles). Sample 090719MK95BHA (half-shaded star) reveals a lower radiogenic excess for all noble gases and is consistent with addition of a minor air component introduced during sampling (see Methods). Error bars represent analytical uncertainty of 1σ and are typically smaller than the plotted symbols.

In the subsurface, noble gases have three potential sources; Air-Saturated Water (ASW), the mantle, and in situ radiogenic production in the crust through radioactive decay of U, Th, and K producing noble gases such as ^4^He, ^21^Ne, ^22^Ne, ^40^Ar, and ^131–136^Xe^30–32^. There is no evidence of a significant mantle input at Moab Khotsong, consistent with previous studies^17,18^. The Moab Khotsong noble gas samples show highly radiogenic ratios with a He R/Ra of 0.02 (where Ra represents the air ^3^He/^4^He ratio of 1.4 × 10^−6^), ^40^Ar/^36^Ar ratios of up to 49,000, and the distinctive elevated radiogenic ^21^Ne/^22^Ne ratios found to date only in hydrogeologically isolated fluids in the Precambrian crust^2,14,18,33,34^ such as the Kidd Creek billion-year-old fluids^2,14^ (Fig. 1).

**Fig. 2:**
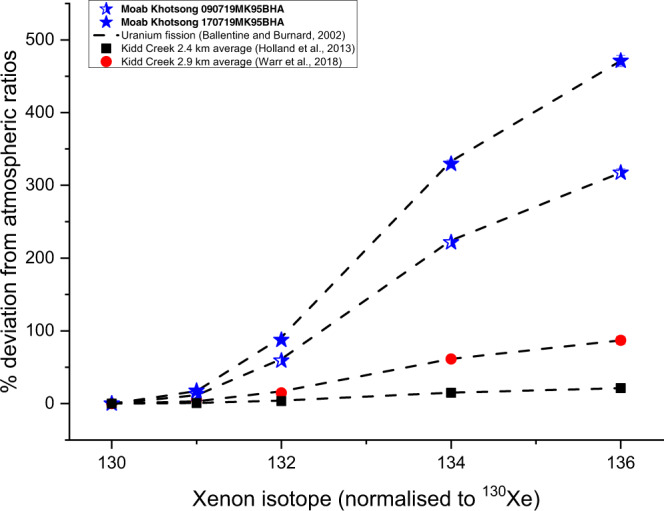
^131–136^Xe isotopic deviations from atmospheric ratios (expressed as percentages). Both Moab Khotsong samples (blue stars) reveal excesses over four times greater than those observed at Kidd Creek and in fluid inclusions at Mt Isa^34^, where the highest excesses previously reported were found, as highlighted in Supplementary Table 2. Published Kidd Creek values^2^ are plotted for context (black squares and red circles). The dashed lines correspond to the production of Xe via uranium fission based on the measured ^136^Xe excess and confirm that uranium fission is the primary source of the heavy Xe isotopic excess in the Moab Khotsong brine. Sample 090719MK95BHA (half-shaded star) reveals a lower radiogenic excess for all noble gases and is consistent with a minor residual air component introduced during sampling (see Methods). Error bars represent analytical uncertainty at 1σ and are smaller than the plotted symbols.

Xe signatures show that ratios of ^131–136^Xe/^130^Xe are the highest observed in fracture fluids, over four times greater than those reported for Kidd Creek^2^, and are consistent with Xe produced by uranium-derived fission added to an initial atmospheric component dissolved in water (ASW) (Fig. 2).

### ^86^Kr – an emerging tracer

Remarkable deviations from ASW like those observed in Ne (Fig. 1) and Xe isotopes (Fig. 2) are also apparent in the ^86^Kr/^80^Kr ratio. Excess Kr (meaning ^86^Kr added in the crust due to radiogenic processes) is calculated as Eq. (1):

1 $${}^{86}{Kr}^{\ast }=\big[{}^{86}{Kr}_{FF}\big]-\big({}^{86}Kr/{}^{80}{Kr}_{ASW}\times \big[{}^{80}{Kr}_{FF}\big]\big)$$

where * denotes radiogenic excess, ASW denotes the initial ratio in Air-Saturated Water, and FF represents the calculated fracture fluid concentrations (see Methods). For sample 170719MK95BHA, the ^136^Xe* concentration of 3.9 ± 0.1 × 10^−9^ cm^3^/cm^3^ of fracture fluid (Supplementary Table 3) corresponds to an ^86^Kr/^80^Kr ratio 7.2% greater than ASW, and a calculated ^86^Kr* concentration of 6.8 ± 0.3 × 10^−10^ cm^3^/cm^3^ of fracture fluid. This radiogenic ^86^Kr excess in groundwater at this locality is sufficiently high that this tracer can now be resolved outside of analytical uncertainty. Together, these heavy noble gas signatures from Kr and Xe at Moab Khotsong indicate these deep fracture fluids are enriched in the highest concentrations of radiogenic products reported to date.

For sample 170719MK95BHA, an in situ ^86^Kr*/^136^Xe* production ratio of 0.177 ± 0.010 is calculated, providing excellent agreement with reported production ranges associated with spontaneous ^238^U fission of 0.125–0.182 (average = 0.16 ± 0.02)^30^. Importantly, the in situ ^86^Kr*/^136^Xe* production ratio for sample 090719MK95BHA agrees as well (0.172 ± 0.008). While 090719MK95BHA has a residual atmospheric component introduced during sampling, this does not affect the ^86^Kr*/^136^Xe* ratio, as ^86^Kr* and ^136^Xe* would be diluted proportionally. The radiogenic ^86^Kr excess identified in these fluids, the ^238^U fission origin for observed ^131–136^Xe* (Fig. 2), and the agreement of ^86^Kr*/^136^Xe* with in situ production ratios, all identify an environment in which the heavy noble gas signatures are dominated by radiogenic processes to an extent never previously identified.

### Billion-year fluid residence times

The highly radiogenic-environment at Moab Khotsong demonstrated by the ^86^Kr* and ^131–136^Xe* excess has important implications for estimating fluid residence times. Starting with the simple assumption of average host rock U, Th, and K concentrations for the region of 2.33 ppm, 10.9 ppm, and 1.91%, respectively^10,35^ (see Methods), and total noble gas release from the host matrix, the apparent residence times calculated for ^4^He*, ^21^Ne*, ^40^Ar*, and ^136^Xe* for the Moab Khotsong fluids vary significantly. Estimates range from 1.03 Ga (^4^He*), 1.36 Ga (^21^Ne*), 1.20 Ga (^40^Ar*) to 4.11 Ga (^136^Xe*), with the apparent Xe-derived residence time unrealistically far in excess of the geologic setting (Supplementary Table 5). Such discordant residence times derived from the different noble gases indicate that simple assumptions of average regional crustal U, Th, and K concentrations are insufficient to describe the system, in particular the high ^136^Xe* excess. The high concentrations of U within this gold-uranium mine setting would have had a major impact on the accumulation of ^4^He*, ^21^Ne*, ^86^Kr*, and ^136^Xe*, but do not affect residence time estimates based on ^40^Ar*, as the production of ^40^Ar is exclusively derived from decay of ^40^K^30^. Thus, although apparent ages are shown for completeness for all four noble gas systems (Supplementary Table 5), and alternative frameworks are considered (see Methods), the most reasonable and conservative estimate of residence times at Moab Khotsong must be the estimate of 1.2 Ga based on ^40^Ar* (see Methods). The dependence and sensitivity of this residence time on the key parameters of radionuclide concentration, noble gas release, porosity, and rock density are fully evaluated in the Methods. The U concentrations that can reconcile the difference between the conservative ^40^Ar* estimate of 1.2 Ga and ^136^Xe* estimates can be calculated, and the ^40^Ar* and ^136^Xe* estimates reconcile for a local U concentration of 19.8 ppm. Such an enhancement above the average values observed elsewhere in the Central Rand Group is consistent with the elevated uranium concentrations in the mining area in general^26,36,37^, and even higher local U values of 309–2847 ppm^29^ reported for the proximal U-rich Vaal Reef conglomerate being extracted at the mine.

To date, deep fracture waters with more than a billion-year residence time had only been identified at Kidd Creek on the Canadian Shield^2,14^. The 1.2 Ga estimate calculated here for Moab Khotsong confirms that the Kidd Creek system is not unique, and most importantly demonstrates that on a global scale the deep crystalline rocks of the Earth’s Precambrian cratons can preserve settings wherein ancient fracture fluids rich in products of water-rock reactions (including both serpentinization and radiolysis)^6,11,12,19^ can be sustained on a geologic timescale. The noble gas residence times and confirmed ^86^Kr* tracer revealed in this study provide a search strategy for identifying ancient fracture water systems around the world where additional research on the deep subsurface hydrosphere should be focused.

### Diffusive loss of the lighter elements

In addition to critical constraints on fracture water residence times from the heavier noble gases, the light noble gases in particular provide process-level information on transport within the deep fracture networks. Although the residence time estimates from ^4^He* and ^21^Ne* at first glance apparently agree within uncertainty with the value of 1.2 Ga derived from ^40^Ar* (Supplementary Table 5), the abundant evidence of high radiogenic input in this environment attested to by the ^86^Kr* and ^136^Xe* require that the impact of high U backgrounds on ^4^He* and ^21^Ne* data also be examined closely.

**Fig. 3:**
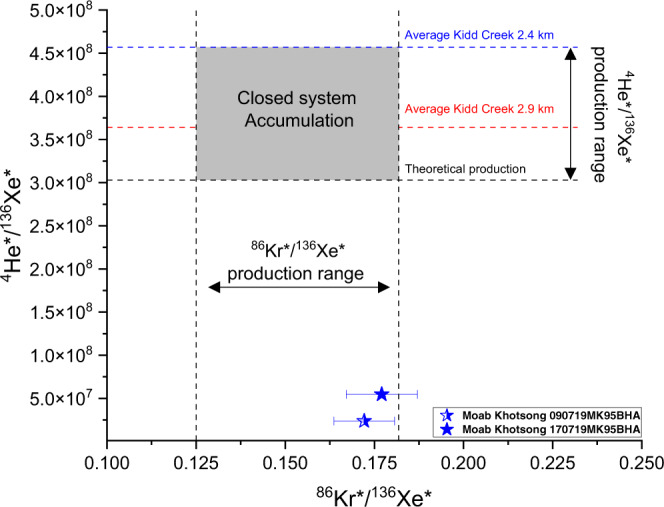
Radiogenic excesses of ^4^He*, ^86^Kr*, and ^136^Xe* for Moab Khotsong brine compared to production ratios, based on predicted values from uranium decay. The ^86^Kr*/^136^Xe* production range is shown between the vertical black hatched lines, and the theoretical ^4^He*/^136^Xe* (between horizontal black hatched line) from Ballentine and Burnard^30^. For additional context, average ^4^He*/^136^Xe* production ranges are plotted for fracture fluids from two depths at Kidd Creek, Canada (blue and red hatched lines), which is considered representative of a closed system given the good agreement between ^4^He*, ^21^Ne*, ^40^Ar*, and ^136^Xe*-derived residence times^2,14^. The shaded grey box then suggests the space that defines a closed system. Both Moab Khotsong samples (blue stars) reveal significant depletion in ^4^He*/^136^Xe* with respect to closed system accumulation, but retain ^86^Kr*/^136^Xe* ratios in line with in situ fissiogenic production from uranium. As atmospheric addition serves to reduce ^86^Kr* and ^136^Xe* proportionally, both samples preserve in situ radiogenic ratios (i.e. this ratio is insensitive to air contamination during sampling seen for sample 090719MK95BHA). Such a pattern of depletion in the light noble gases ^4^He* (and ^21^Ne*) with respect to their heavy noble gas counterparts is consistent with diffusive loss. Error bars incorporate propagated analytical uncertainty of 1σ.

Fig. 3 demonstrates that, as expected for two elements that would be proportionally impacted by U content, ^86^Kr*/^136^Xe* production ratios for the Moab Khotsong samples are within the predicted in situ production range and hence consistent with in situ accumulation, as discussed previously. In contrast, Fig. 3 demonstrates that the ^4^He*/^136^Xe* ratios are significantly lower than would be predicted based on expected in situ production ratio. As ^21^Ne* and ^136^Xe* are produced proportionally to ^4^He* in the crust by radiogenic processes, ^21^Ne*/^136^Xe* ratios should also be independent of radioelement concentration^14,30^, but Supplementary Fig. 2 demonstrates that both ^21^Ne*/^136^Xe* and ^4^He*/^136^Xe* ratios (Fig. 3) are substantially lower than theoretical production would predict. Both of the light noble gases reveal instead a pattern consistent with elemental fractionation via loss of the light noble gases relative to the less diffusive heavy noble gases. Diffusive loss of the light noble gases can account for the lower ^4^He*- and ^21^Ne*-based residence times compared to those based on ^40^Ar* and a U concentration of 19.8 ppm. Fig.3 shows that this pattern of He (and Ne; Supplementary Fig. 2) loss contrasts with the hydrogeologically isolated Kidd Creek fracture network, where the He-Xe ages at comparable depths all agree within uncertainty^2,14^. The data demonstrate that while the Moab Khotsong system preserves ancient fluids on a billion-year timescale, and, like Kidd Creek is hydrogeologically closed to mixing with younger fluids, diffusive transport is still possible and a significant degree of diffusive gas loss has occurred, preferentially impacting the light noble gases.

The degree of diffusive loss of He and Ne can be quantified by incorporating the 1.2 Ga residence time derived from ^40^Ar*. As noted, this residence time estimate is unaffected by regional variations in U concentrations. Further, Ar is much less affected by diffusive loss through the crust as its diffusion coefficient is lower by a factor of up to 460 relative to He and Ne^30,38^. Consequently, this is the most robust estimate of fluid residence times at Moab Khotsong. Using a 1.2 Ga residence time and the estimated local U concentration of 19.8 ppm (derived from the ^136^Xe* calculation above) suggests that concentrations of ^4^He* and ^21^Ne* are only 18 and 25%, respectively, of what would be predicted based on local production ratios. Loss of 82% of the produced ^4^He* and 75% of the ^21^Ne* is both consistent with slow, long-term, diffusive loss from low porosity crystalline rocks, and with the preferential loss of He with respect to Ne based on their relative diffusion coefficients^30,38,39^. The consistency between the observed ^131–136^Xe* and expected fissiogenic production ratios from U (Fig. 2), coupled with the ^86^Kr*/^136^Xe* production matching predicted production ratios (Fig. 3), confirm negligible diffusive loss of both Kr and Xe. For simplicity, this model considers slow, long-term progressive diffusion over long geologic time. However, regional tectonic events, as discussed in the following section, may have resulted in periods of enhanced rate of loss of these light elements. Overall, the noble gas data reveals that while Moab Khotsong is, like Kidd Creek, preserving ancient fluids on unprecedented geologic timescales and hydrogeologically closed to mixing with younger fluids derived from the surface photosphere, the system is open to diffusive loss of the lighter components. This therefore highlights the wealth of process information on fluid residence time and transport available from the suite of light to heavy noble gases.

## Discussion

Crystalline rock-hosted fracture fluids in Precambrian cratons globally create settings where products of water-rock reaction, including radiolysis and serpentinization, can accumulate and preserve habitable environments on timescales much longer than the <200-million-year-old oceanic crust. Most studies to date have focused on fracture systems still in connection with the surface on timescales of up to tens of millions of years^16,17,40^. Considerable attention too has focused on an example of extended hydrogeologic isolation at Kidd Creek in Canada where concordant residence times indicate isolation on a 1–2-billion-year timescale^2,14^. Understanding the processes controlling isolation versus connection of deep fracture networks, and the implications for the nature and distribution of microbial life hosted within these environments, requires targeted investigation of sites along the spectrum from open to closed. To date, the Witwatersrand Basin has provided the largest and most diverse spectrum of continental sites, ranging from those with meteoric groundwaters less than a decade to few thousand years old^41^, to sites dominated by paleometeoric water that recharged from surface to >3 km depth up to 100 Ma years ago^17,18,21,36^. Data compiled by Warr et al.^9^ for these environments confirm that δ^18^O and δ^2^H data for the less saline waters plot on, or near to, the GMWL, consistent with varying degrees of penetration and mixing of paleometeoric waters. Unlike the geologic setting of Kidd Creek, which is a well-preserved section of the Archean crust^14,19^, the Witwatersrand Basin underwent a major impact event at 2.02 Ga^42^. At another major impact crater, the Sudbury Basin in Canada, 313–544 Ma fluids at depths of 1.4 to 1.7 km were attributed to the regional effects of fracturing related to the impact that enabled greater penetration of younger fluids into the crust than observed at Kidd Creek^2^. The spectrum of paleometeoric waters penetrating to great depth at many sites in the Witwatersrand Basin may similarly have been facilitated by the impact, and late-stage intrusive processes, and augmented by late-stage plateau elevation^17,18,21,36,41^.

The data from the newly investigated site at Moab Khotsong in this study extends the spectrum of sites in South Africa by demonstrating the presence of an ancient billion-year-old brine end-member. Previously published δ^18^O and δ^2^H isotope data for Moab Khotsong are among the most elevated above the GMWL ever reported^9^ (Supplementary Table 1). This paper quantitatively establishes the Ga-residence times and confirms the ^86^Kr* and noble gas signatures that demonstrate the unprecedented radiogenic content of the Moab Khotsong fracture fluids. Importantly, the data demonstrate that while no hydrogeologic mixing with younger fluids is occurring, diffusive transport is an important process controlling loss of He and Ne relative to the heavy noble gases (Ar, Kr, Xe). This study provides critical insight into the degree to which deep fracture networks represent virtual time capsules in the crust, as at Kidd Creek, or whether, as at Moab Khotsong, the system may be hydrogeologically closed to penetration and mixing with surface-derived fluids, but still open to diffusive loss of light elements on long geologic timescales. Specifically, Moab Khotsong confirms that significant loss of more mobile fluid components (e.g. He, Ne, and potentially H_2_) can occur even where fluids have Ga residence times. Though He flux from the basement crust is commonly considered for overlying geologic systems^30,39,43–45^, outward fluxes of H_2_, CH_4_, and other light molecules produced in the basement^3,4,6,11,46^ are less common^44^. Given that deep cratonic systems are estimated globally to produce H_2_ and He on the same order of magnitude as the oceanic crust^3,4,30^, long-term flux from ‘more open’ systems such as Moab Khotsong into overlying lithologies may additionally provide a deep flux of H_2_ too within significant volumes of the crust over planetary timescales. Conversely, where diffusive fluxes are minimal (e.g. Kidd Creek), these deep fracture fluids may exist in long-term isolation^11,19,47^. With respect to noble gas budgets, the global production of noble gases in these deep crustal settings has been recently revealed to be comparable to production from the oceanic crust and mid-ocean ridges^4^. This paper demonstrates, through a process-based investigation applying the full suite of noble gases, that deep continental settings have the capacity to accumulate gases and fluids on up to billion-year timescales. Distinguishing between diffusive flux out of the subsurface, and/or geologic accumulation of these components, is critical to evaluating the significance of potential sites for geologic storage^16,39,45,48^, and in the context of formation of economic-grade noble gas reservoirs in a world rapidly running short of helium^49^.

This study demonstrates how, in these radiogenically-dominated environments, even conservative noble gas signatures can be substantially different compared to average crust, and underscores the necessity to re-evaluate our understanding of radiogenic processes and end-members, both for conservative noble gases, and for reactive molecules such as H_2_, SO_4_ or acetate produced by radiolysis in addition to other water-rock reactions such as serpentinization^3,11,19^. Such radiogenically enriched- environments will inevitably also impact assumptions about the in situ production processes, rates and background levels for radiogenic-linked groundwater dating tracers such as ^3^H, ^14^C, ^36^Cl, and ^81^Kr, in addition to radiogenic noble gases. The findings of this study underscore the need to expand understanding of key radiogenic processes in the crust, both continental and marine, to validate quantitative models of fluid age and transport critical to understanding the degree of isolation, or degree of connection, of the subsurface to surface processes. Constraining radiogenic processes and their associated fluxes will provide refined habitability models of the subsurface, both for the Earth and on other solar system bodies, where habitability driven by radiolysis a major driver of current investigation^12,44,50–52^.

## Methods

### Sample collection

Two noble gas samples were collected in 2019 from a borehole located at 2.9 kmbls in Moab Khotsong Mine, South Africa, following methods described in previous studies^2,14,17^. Briefly, gas tight Tygon tubing was connected to a sampling valve on an Inconel U-tube/packer device^53^. This device had been installed into the borehole to collect fluids entering the borehole from intersecting fracture fluid networks. A downhole pressure/temperature sensor (GEO PSI, Calgary, AB T2E 8Z9, Canada) indicated that the in situ fluid pressure was 10.2 MPa and temperature was 54 °C. The sampling valve was then opened and the pressurized fluids flowed through a gas stripper to separate the gas and water phases. Gas samples were collected in triplicate per standard sampling techniques in refrigeration-grade, internally polished, 3/8” diameter copper tubes. Prior to sample collection, gas flow rates were measured and, based on this, the sampling apparatus was flushed a minimum of 6 times the internal volume to ensure the collected gas samples were representative of the fracture network. Once flushing was complete, the copper tubes were cold-welded shut in sequence using a hydraulic crimping device.

For gas compositional analysis, samples were collected in pre-evacuated 160 mL borosilicate glass serum vials containing 100 μg of saturated HgCl_2_ using a gas-tight syringe with a 22-g syringe needle following established methods developed for sampling these gases^6,54^.

### Noble gas analysis

The noble gas samples were extracted, purified, and measured at the Noble Laboratory, University of Oxford using existing protocols^55^ previously modified for analyzing highly radiogenic gas samples^2,17^. These published protocols are outlined here and were used for samples, procedural standards and blanks. First, samples were expanded to a calibrated volume containing a 1000 Torr Baratron at high vacuum, and the pressure and temperature was recorded. Next the sample was introduced to activated titanium sponge (~30 g) held at 1223 K (950 °C) for 15 min to chemically remove the majority of the active gases (e.g., N_2_, CH_4_, C_2_H_6_). The titanium sponge was then allowed to cool while still exposed to the sample for a further 30 min to allow removal of H_2_. An additional purification step was then applied to the sample using a combination of a hot (SAES GP-50) and cold (SAES NP-10) getters for a further 15 min to remove any residual active gases remaining in the sample. Next, the purified sample was passed through a series of calibrated, liquid-helium-cooled cryogenic traps of decreasing temperature to first isolate and remove any water vapour (stainless steel trap held at 180 K), then trap the argon, krypton and xenon components (stainless steel trap held at 33 K) and lastly trap helium and neon (charcoal trap held at 15 K). Each of these three trapping stages lasted 15 min^2,17,55^.

For the helium analysis, the gas was first released by heating the charcoal trap to 31 K for 15 min. The released helium was then split using calibrated volume expansions and an aliquot was introduced and measured using a ThermoFisher Scientific HELIX SFT. Peak centres were defined manually and the two isotopes of helium, ^3^He and ^4^He, were measured simultaneously on an electron multiplier and on a Faraday respectively.

Prior to measuring neon, the residual helium first needed removal from the cryogenic trap. This was efficiently achieved following the published method of Warr et al.^2^ by cycling the charcoal trap to 50 K for 15 min then back down to 31 K for a further 15 min. The gas was then expanded to the manifold and pumped, during which the charcoal trap was isolated. This cycle of heating, cooling and static pumping was repeated two additional times and ensured any residual helium in the trap volume was efficiently removed without affecting neon. With the residual helium removed the neon component was then released from the charcoal trap by heating to 90 K for 15 min. After this, a calibrated volume was inlet into a ThermoFisher Scientific ARGUS VI for measurement. During each analytical cycle ^20^Ne and ^22^Ne were measured on Faraday cups and ^21^Ne was measured on a multiplier. ^40^Ar and CO_2_ were additionally measured during this analysis in order to correct for any minor ^40^Ar^++^ interference peaks^55^. Once neon analysis was complete the charcoal trap was opened to the vacuum pump and heated to 300 K to release and remove any remaining sample.

For argon, the stainless-steel trap was initially heated to 200 K for 15 min to release all trapped noble gases (Ar-Kr-Xe) and a small aliquot of this gas was taken. The trap was then cooled back down to 52 K to allow re-trapping of the heavy noble gases. While this re-trapping was taking place, the argon aliquot was expanded and diluted using calibrated volumes and then inlet to the ThermoFisher Scientific ARGUS VI which measured ^40^Ar on a Faraday cup and ^36^Ar and ^38^Ar on an electron multiplier via peak-jumping.

Prior to measuring krypton and xenon, the residual argon also required removal from the cryogenic trap. As with the helium removal, this was achieved following published methods of heating, re-cooling and pumping specifically developed for analyzing highly radiogenic samples^2,17^. For this, the stainless-steel trap was heated for 5 min from 52 K to 70 K and then cooled back down to 52 K, to allow argon release and full re-trapping of krypton and xenon followed by 2 min dynamic pumping to remove the argon. After 10 cycles the stainless-steel trap was heated to 200 K to ensure full release of all krypton and xenon. The sample was then inlet into the ThermoFisher Scientific ARGUS VI where all krypton and xenon isotopes were measured in one analysis using peak jumping with ^124^Xe and ^126^Xe measured on the electron multiplier and all other isotopes were measured using Faraday cups.

Per standard procedures for noble gas analyses, samples were blank-corrected and normalized using full procedural air standards and blanks which were purified and analyzed using the same protocols as the samples outlined here to allow for direct comparison. All blank subtractions and ^40^Ar^++^ corrections were <1% on the measured noble gas isotopes. The standard was collected in University Parks, Oxford, UK, under known environmental conditions (15.7 °C, 28% humidity and 1032 mBar pressure). All measured noble gas concentrations and ratios are provided in Supplementary Tables 1 and 2 respectively.

### Compositional analysis

The gas compositions in Supplementary Table 4 were determined at the Stable Isotope Laboratory at the University of Toronto, following established methods^46,54^. First, samples were injected into a Varian 3400 Gas Chromatograph (GC) coupled with a thermal conductivity conductor (TCD) to determine the inorganic gas components (H_2_, He, N_2_, Ar, and O_2_). H_2_, He and N_2_ were measured by passing an injected sample through a Varian Molecular Sieve 5 A PLOT fused silica column (0.53 mm OD x 25 m) using argon as a carrier gas. To separate out these components prior to being measured via the TCD, the oven temperature was initially set to 10 °C for 600 s after which it was increased to 80 °C at a rate of 25 °C/60 s. For the Ar and O_2_ analysis the same column was used with helium as a carrier gas and the oven temperature was held at −10 °C to ensure complete separation between Ar and O_2_ within the column prior to being measured via TCD. To analyse the samples for their hydrocarbon content (CH_4_, C_2_H_6_, C_3_H_8_ and C_4_H_10_) a Varian 3380 GC coupled with a flame ionization detector (FID) was used. Similar to the inorganic gas analyses, the hydrocarbons were separated within the GC by passing each injected sample through a J&W Scientific GS-Q column (0.32 mm OD x 30 m) using helium as a carrier gas. For separation of these gases the column was initially held at 60 °C for 150 s after which it was increased to 120 °C at a rate of 5 °C/60 s. All compositional analyses were run in triplicate and mean values calculated. The analytical uncertainty on compositional analyses is ± 5% by volume.

### Quantifying air contamination

From the two samples analyzed for the 23 noble gas isotopes, sample 090719MK95BHA had reduced radiogenic noble gas ratios and concentrations with respect to the second sample (170719MK95BHA), consistent with introduction of a minor air component (1–3%) during sampling. This air contamination during sampling affects radiogenic to non-radiogenic noble gas ratios (e.g. ^40^Ar/^36^Ar) and radiogenic excesses and therefore precludes calculation of representative residence times being derived for this sample. However, radiogenic/radiogenic ratios (e.g. ^86^Kr*/^136^Xe*) are unaffected as the air dilution effect impacts both elements proportionally.

### Quantifying degassing and concentrations

In order to determine the concentrations of the noble gases in the fracture fluids prior to sampling, the approach of Holland et al., Warr et al., and Heard et al., were used^2,14,17^. As per standard practice in noble gas studies for fluids with temperatures and salinities similar to these types of system^2,14,17,36^, an air-saturated water (ASW) noble gas content of seawater at 20 °C was taken as the starting point. Comparing the measured elemental ratios to this starting composition (Supplementary Fig. 1), the non-radiogenic noble gas isotopes reveal that the gas samples have been fractionated and preferentially enriched in the light noble gases (e.g. Ne) relative to their heavy counterparts and is consistent with partial degassing of fluid with a seawater like noble gas composition. Standardly this elemental fractionation is attributed to incomplete degassing of the water phase (i.e. solubility-driven fractionation) and is consistent with the observed low gas and water flow rates during sampling^2,17,55^. This can be estimated by applying a least-squares approach comparing the measured elemental non-radiogenic noble gas ratios (^20^Ne/^36^Ar, ^80^Kr/^36^Ar, and ^130^Xe/^36^Ar) with modelled, step-wise Rayleigh degassing of the water phase under sampling conditions (60 °C, 6 M salinity) using published noble gas solubilities and Eq. (2).

2 $$S=	\,{\left(\frac{{\;\!}^{20}Ne/{\;\!}^{36}A{r}_{degas}-{\;\!}^{20}Ne/{\;\!}^{36}A{r}_{samp}}{{\sigma }_{samp}}\right)}^{2}+{\left(\frac{{\;\!}^{80}Kr/{\;\!}^{36}A{r}_{degas}-{\;\!}^{20}Kr/{\;\!}^{36}A{r}_{samp}}{{\sigma }_{samp}}\right)}^{2}\\ 	+{\left(\frac{{\;\!}^{130}Xe/{\;\!}^{36}A{r}_{degas}-{\;\!}^{130}Xe/{\;\!}^{36}A{r}_{samp}}{{\sigma }_{samp}}\right)}^{2}$$

where *S* represents the sum of the squared residuals, degas and samp indicate the isotopic ratio for a given degassing step in the stepwise degassing and the observed ratio in the sample respectively and σ is the associated measurement uncertainty for each measured ratio. The degassing ratios are determined by progressively modelling the evolution of a gas phase as 0.1 cm^3^ of gas exsolves from 1000 cm^3^ of fluid, to simulate open degassing under low gas-to-water ratios during sampling in accord with previous approaches^2,17,55^. Fit is assessed by comparing the modelled noble gas ratios in the gas phase to measured ratios with the lowest S value representing the best fit of the model to the data. This approach, when applied to sample 170719MK95BHA, resulted in a best-fit estimated degassing of 61% (He), 59% (Ne), 42% (Ar), 31% (Kr), and 25% (Xe) from the initial ASW composition proposed here.

In order to determine the initial radiogenic noble gas content of the water, the approach of Warr et al.^2^ was used to correct for incomplete degassing of the water phase. Briefly, the relative enrichment/depletion factor over ASW values was determined using ^20^Ne, ^36^Ar, ^80^Kr and ^130^Xe concentrations for each noble gas and all other isotopes are corrected accordingly based on their measured ratios. As He has no non-radiogenic isotope, the Ne degassing coefficient was applied given their comparable solubilities as per previous studies^2,17^. These radiogenic noble gas concentrations are provided in Supplementary Table 3.

### Calculating fluid residence times

The noble gas-derived fracture fluid residence times were calculated based on a closed system with radiogenic ingrowth model in line with previous widely published methods^2,14–17^ and are summarized as follows. First, the radiogenic excesses of ^4^He, ^21^Ne ^40^Ar and ^136^Xe per cm^3^ of fracture fluids were multiplied by the porosity to calculate the radiogenic excess concentration per cm^3^ of rock. A porosity of 0.9% (±0.4%) was taken for this lithology based on depth porosity models^2–4,56^ and in line with previously published values for the Witwatersrand Basin^10,57^. This was then multiplied by the density (2.85 g/cm^3^)^17,36^ to derive the radiogenic excess per gram of rock. For He and Ar this was incorporated into Eqs. (3) and (4):

3 $${\,\!}^{4}H{e}^{\ast }=8\times [{\,\!}^{238}U]\times ({e}^{{\lambda }_{238}t}-1)+7\times [{\,\!}^{235}U]\times ({e}^{{\lambda }_{235}t}-1)+6\times [{\,\!}^{232}Th]\times ({e}^{{\lambda }_{232}t}-1)$$

4 $${\,\!}^{40}A{r}^{\ast }=0.105\times [{\,\!}^{40}K]\times ({e}^{{\lambda }_{40}t}-1)$$

where ^4^He* and ^40^Ar* are the radiogenic excesses produced over time *t* in yr, [^238^U], [^235^U] [^232^Th], and [^40^K] represent the concentration of ^238^U, ^235^U, ^232^Th, ^40^K respectively in atoms/g, 8, 7 and 6, represent the total ^4^He production via each decay chain, 0.105 is the fraction of ^40^K which decays to ^40^Ar, and *λ* are the respective decay constants in yr^−1^. For the ^21^Ne* and ^136^Xe* residence times the ^4^He*/^21^Ne* and ^4^He*/^136^Xe* production ratios of 3.95 × 10^7^ and 3.033 × 10^8^ were applied to Eq. (3) based on established production ratios for Xe^2,14,30^ and a site-specific ^4^He*/^21^Ne* per the following section. To ensure all residence time estimates are conservative, 100% noble gas release from the host rock mineralogy was considered for these models as per the method of Holland et al.^14^, in line with previous noble gas accumulation models considered for the Witwatersrand Basin^9,17,18,21,36^. For the radioelement concentrations a regional average was used as fracture networks are much larger than the thickness of individual stratigraphic units^17,58^. A regional host rock concentration for the Witwatersrand Basin of U, Th, and K of 2.33 ppm, 10.9 ppm and 1.91% respectively was used initially per previously published studies for the Central Rand Group^10,17,35^. This approach resulted in apparent residence times of: 1.03 ± 0.47 Ga (^4^He*), 1.36 ± 0.62 Ga (^21^Ne*), 1.20 ± 0.54 Ga (^40^Ar*), and 4.11 ± 1.87 Ga (^136^Xe). Using a site-specific ^4^He/^136^Xe of 3.992 × 10^8^ after the calculations of Heard et al.^17^ only slightly changes this ^136^Xe value by 15% to 4.74 ± 2.15 Ga, within error of the original estimate and confirming the validity of the ratio used here. Although U concentrations are elevated in the region (main text) no corresponding enrichment in K concentration has been observed where U concentrations are particularly elevated^29^. Consequently, the K-dependent (and U-independent) ^40^Ar* residence times provide the most robust estimate for these fluids (see main text). Nonetheless, to further evaluate robustness of the ^40^Ar*-derived residence time estimate, a lower K concentration of 1.47% based on published values for the West Rand Group^10^ was evaluated. This slightly increases the residence time to 1.44 ± 0.65 Ga, but this remains within the calculated uncertainty of the original estimate. Likewise, changing bulk rock density has a minimal effect; using a lower value of 2.70 g/cm^3^ from elsewhere in the Witwatersrand Basin^57^ results in residence time estimates only increasing by 4% to 1.24 ± 0.56 Ga which is again well within uncertainty. Given the critical role of porosity in these residence time calculations as outlined in previous papers^2,14^, this parameter was also evaluated. For crystalline rock bulk fracture-induced porosity rather than matrix porosity is the most appropriate parameter to determine flow in fractured hydrogeologic environments^59^. Reported bulk porosity values measured from crystalline rock globally reveal a range of 0.1–2.1%^60–64^ which is consistent with the published range of 0.1–2% reported for typical crystalline rocks in the Witwatersrand Basin^10,57^. This range is also consistent with modeling approaches for porosity changes as a function of depth in the crust which estimate an average porosity of 0.96% for the upper 10 km of the crust^3,56^. The porosity value applied here of 0.9% is derived from this depth-dependent approach and is within the published ranges for these settings. Additionally, for this study an uncertainty of ±0.4% is applied, following previous approaches^2,14,17^, to reflect variability in this key parameter in line with the natural variability that may exist on regional-global scales. This therefore provides a spectrum of residence times which reasonably incorporates natural variation in these types of setting. Lastly, although negligible diffusive loss of ^40^Ar* is considered here in contrast to ^4^He* and ^21^Ne*, given the difference in diffusion coefficients (main text)^30,38^ any modest loss would slightly increase the ^40^Ar* subsurface residence times presented here, again, highlighting the conservative nature of this age estimate. Consequently, the residence time of 1.20 ± 0.54 Ga presented here also represents the most robust estimate for ^40^Ar*. As is typically the case for radiogenic noble gas-derived residence time estimates^2,3,14,17^ the variability in porosity estimates represents the dominant source of uncertainty.

### Uranium-rich Ne production

While ^4^He*, ^86^Kr*, and ^131–136^Xe* are all directly produced via uranium decay, ^21,22^Ne* are produced in the subsurface principally through the interaction of radionuclides and the host rock through the following two reactions: ^18^O(α,n)^21^Ne, and ^19^F(α,n)^22^Ne^18,30^. Consequently mineralogy (specifically the localized O/F ratio in the vicinity of U-bearing minerals) can significantly affect subsurface Ne production^18,30^. At Moab Khotsong, the U-rich Vaal Reef is the primary U-bearing lithology mined^29^. Studies of the Vaal Reef mineralogy have revealed that the uranium in this deposit is principally concentrated in three minerals: uraninite, coffinite, and brannerite^29^. In such minerals, the measured production of ^4^He*/^21^Ne* is considerably higher compared to production ratios of 1.0–2.2 × 10^7^ previously applied to other deep subsurface sites in South Africa, Canada and Finland^2,14,16,17^, which all have U concentrations closer to global averages for crystalline rock. Using the ^4^He*/^21^Ne* production ratios of 3.5 × 10^7^ and 4.6 × 10^7^ for uraninite and coffinite, respectively^65^, along with their proportional abundances, a more representative ^4^He*/^21^Ne* production ratio of 3.95 × 10^7^ is applied here to reflect ^21^Ne production in a U-rich environment at Moab Khotsong. This ^4^He*/^21^Ne* production ratio is 2.3× greater than the average crustal production rate^30^ and reflects the notably lower ^21^Ne production rate. This is inferred here to be due to a lower interaction between radionuclides and light elements in the host rock as indicated by decreased neutron fluxes generated from a light element interaction per alpha particle generation for U-rich mineralogies^30,66^.

### Other Kr isotope production

Although ^86^Kr* produced via spontaneous fission of ^238^U additionally produces both ^84^Kr* and ^83^Kr*, the production rates with respect to ^86^Kr* are considerably lower and are 0.13–0.22 and 0.03–0.06 respectively^30^. With ^84^Kr in ASW naturally 3.3× more abundant than ^86^Kr, and with the ^83^Kr production rate being so low, any radiogenic addition to either isotope falls well within analytical uncertainty for these samples. Likewise, although the highly radiogenic environment makes Moab Khotsong a prime candidate for ^81^Kr and ^85^Kr analysis, neither could be measured using conventional noble gas analytical techniques, given their low concentrations and production rates^37^.

### Sensitivity assessment of model outcomes

Although the majority of noble gas studies for these deep subsurface environments typically incorporate in situ long-term production and accumulation^2,9,14–18,21,36,37^, alternative models to incorporating mixing, or addition from ancient metamorphic fluids have also been applied to interpret noble gas data from the Witwatersrand Basin^18,21,23^. This alternative model was additionally considered here for the data set to evaluate the robustness of our findings. In this scenario, instead of calculating the mean residence time through long-term ^40^Ar* release and accumulation, release is exclusively modelled in the context of high temperature hydrothermal activity when temperatures and pressures were sufficiently high (350 ± 50 °C and 1.5–3 kbar)^25^ to facilitate efficient and rapid noble gas release from the host mineralogy^30^. Under such parameters the oldest theoretical fluid component in the Witwatersrand could correspond to the last recorded period of regional metamorphism at 2.1–2.0 Ga^25,67^ and subsequent low temperature release would be considered negligible for ^40^Ar*, in line with previously reported ^40^Ar* mineral ages for the region which approach the last regional metamorphism^67–69^. Low temperature release of He, (and potentially Ne) would remain plausible due to their greater mobility and a more energetic production route for He (α-recoil)^30^. Likewise, Xe (and Kr) may also be potentially be released at low temperature due to recoil loss^70^.

Importantly, even interpreting the noble gas data presented here under this alternative framework, results in only relatively minor overall differences from those described in the main text (only a slight increase from a maximum ^40^Ar* age 1.74 Ga (based on 1.20 ± 0.54 Ga) (main text), to 2.1–2.0 Ga^25,67^), and hence no change in the major conclusions of this paper. Likewise, the observed differences between the light and heavy noble gases (and the associated low ratios – Fig. 3) continue to support long-term light diffusive loss as a major process affecting the Moab Khotsong system. Given that ^4^He* (and ^21^Ne*) are expected to be released at a comparable or greater rate than ^86^Kr* and ^136^Xe*, the estimates of 82 and 75% total loss for ^4^He* and of the ^21^Ne*, would, in this alternative framework, instead represent minimum loss over total production. Lastly, with regards to a local U concentration, assuming a fluid age of 2 Ga and a starting ^136^Xe* concentration of zero (i.e. all derived from in situ radiogenic production), a maximum U value of 9.8 ppm is calculated, not substantially different from the value of 19.8 ppm. This concentration decreases proportionally as the relative percent of ^136^Xe* associated with hydrothermal release is increased from 0%. None of these relatively minor differences though meaningfully affect the fundamental conclusions as described in the main text and hence provide additional support for the robustness of the study’s major conclusions and implications.

Although this model is presented here to provide a sensitivity assessment of the overall findings, it is worth noting within the geologic setting of the Witwatersrand Basin, previously published data from a variety of different sites support the assumption of 100% release for the light noble gases. Specifically, modelled ^4^He* and ^40^Ar* residence times (which consider 100% release for both noble gases and closed system accumulation) are highly consistent over Ka-Ma timescales^17,18^. This concordance is difficult to reconcile within a regional model involving variable mixing between a ^40^Ar*-rich hydrothermal fluid and more modern counterparts, such as is involved in this alternative framework. Further, the alternative framework considers only thermal release of ^40^Ar*. However, K-Ar mineralogical analyses indicate that the fluids associated with this hydrothermal activity likely had initial ^40^Ar/^36^Ar ranges between 274–416^69^ which is consistent with the proposed initial seawater ratio of 295.5 applied here as the starting fluid. Crucially this initial range falls two orders of magnitude below the ratio of 48,617 measured in these fluids (Supplementary Table 2). Thus, a post-hydrothermal (and therefore low temperature) source of ^40^Ar* still is required. Release of noble gases over geologic timescales can additionally occur at low temperatures through both secondary mineralization and associated water-rock reactions^16^ and diffusion into, and along grain boundaries^30^. Related to this, Sleep and Zoback^71^ specifically reported that fracture opening occurs, even in quiescent cratons (such as the setting for this study), on a kilometre scale on a periodicity of 1 to several million years. Even in hydrogeologically isolated, tectonically quiescent settings such as this, over the > 2-billion-year age of this geologic setting, fracture opening and the potential for fracture intersection and fluid mixing, and release of noble gases, is an ongoing process. Consequently, localized, effective progressive degassing of ^40^Ar (and other noble gases) can occur from lithological units in the proximity of fracture networks into the fluids where they accumulate, while more distal mineralogies may retain higher proportions their noble gases. To elaborate and clarify a little further, in all geologic settings, the specific mechanisms of release from the host lithology into the fluids are never instantaneous in the absolute sense, be it diffusion, mineral alteration, or through (micro)fractures and so on. However, as long as the rate of release (Ma) is relatively rapid relative to the overall fluid residence time, (here hundreds of Ma to Ga), this can be reasonably approximated as progressive, on-going release of noble gases from the host rock into the fracture fluid networks, as we apply here. It should be noted though that in fractured rock settings where fluid residence times are considerably lower than these ~Ma release timescales this noble gas-based approach can potentially underestimate calculated residence times. In such scenarios, additional, independent, short-lived radioelement tracers (e.g., ^3^H, ^14^C, ^36^Cl, ^39^Ar) may also therefore be applied to evaluate the release of noble gases into the fluids over these shorter timescales e.g.,^17,36,37,72^. These, or comparable approaches, will provide the basis for essential calibration of existing models and allow for accurate constraints on the shortest residence times that noble gases can determine in these settings. Critically, any of these additional concepts do not significantly affect the principal findings of this study, and the alternative model discussed above instead provides an alternative residence time of 2.1–2.2 Ga that robustly delivers an independent confirmation of the same range of billion-year-old residence times as the main text. Here though, where fluids have hundreds of Ma to Ga residence times, considering 100% release in this context (and zero retention) therefore results in the most conservative estimates of radiogenic noble gas concentrations in the system being considered which correspond to conservative residence time estimates. Due to these above points, the accumulation model with 100% release (as outlined in the main text) is considered the best fit to the noble gas data presented here with 1.2 Ga remaining the most conservative estimate of fluid residence times.

## Supplementary information

Supplementary Information

## Data Availability

The data generated in this study are provided in the Supplementary Information file.
