# Supplementary material for: 86Kr excess and other noble gases identify a billion-year-old radiogenically-enriched groundwater system
Source: Nat Commun. 2022 Jun 30;13:3768. doi: 10.1038/s41467-022-31412-2 (PMC9246980; doi:10.1038/s41467-022-31412-2)
Supplement: Supplementary file 1 — Supplementary Information [file 41467_2022_31412_MOESM1_ESM.pdf]

^{86}Kr excess and other noble gases identify a billion-year-old radiogenically-enriched groundwater system

Supplementary Information

Supplementary Figure 1. Comparing the measured elemental ratios from sample 170719MK95BHA to Air Saturated Water at 20°C

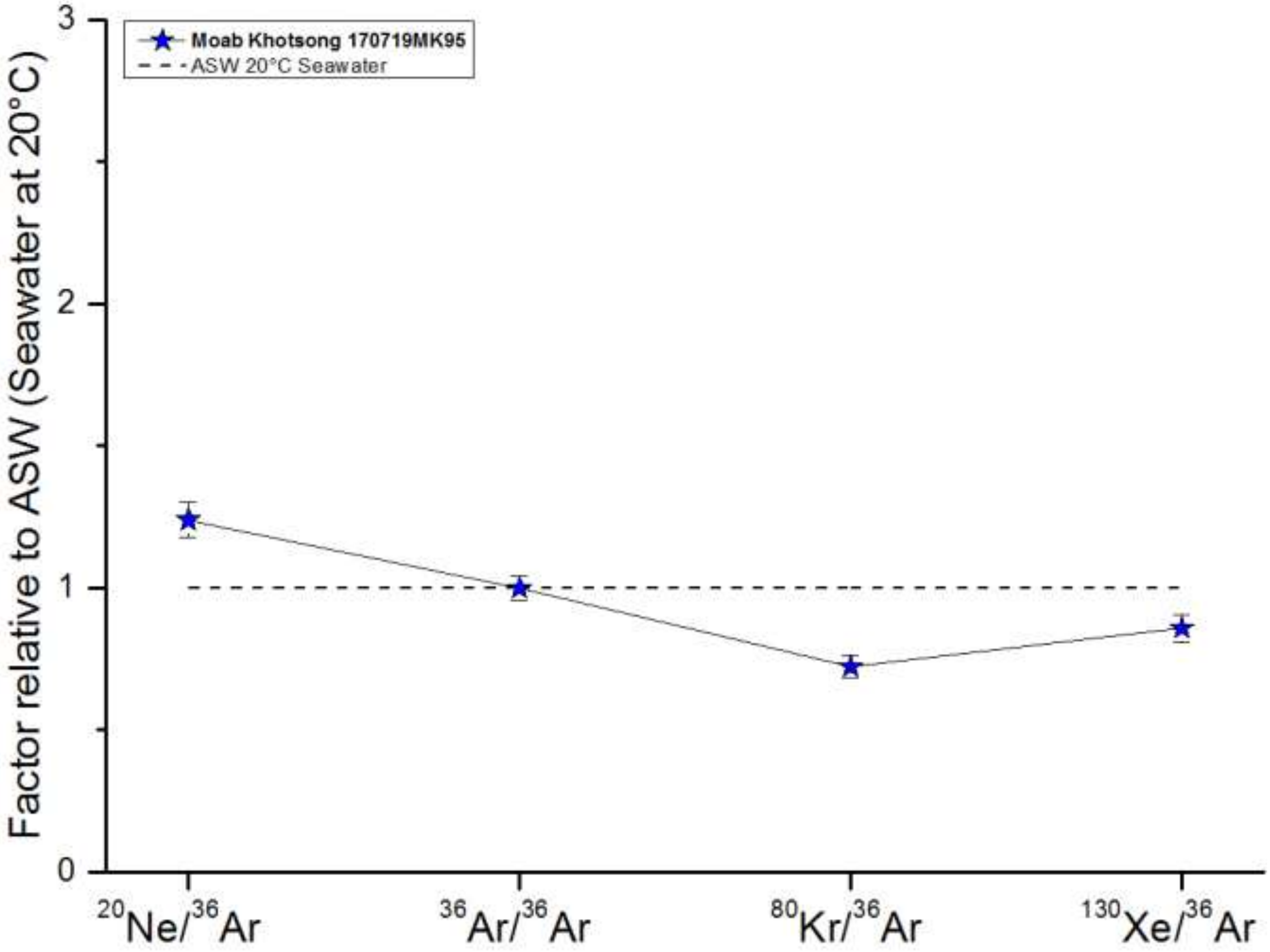

This figure reveals that the non-radiogenic noble gas isotopes (blue stars) have been fractionated relative to Air Saturated Water (ASW) (black hatched line) and preferentially enriched in the light noble gases (Ne) relative to their heavy counterparts and is consistent with partial degassing of fluid with a seawater like noble gas composition (see main text). This fractionation has similarly been observed at Kidd Creek and is due to incomplete degassing of the water phase (i.e., solubility-driven fractionation) consistent with the observed low gas and water flow rates during sampling[2,17,55]. This fractionation during sampling of noble gases is traditionally accounted for when reconstructing initial concentrations[2,4,14,17,30] (see details in Methods). Error bars represent full propagated analytical uncertainty of 1σ.

Supplementary Figure 2: Radiogenic excesses of ^{21}Ne, ^{86}Kr, and ^{136}Xe for Moab Khotsong brine compared to calculated production ratios

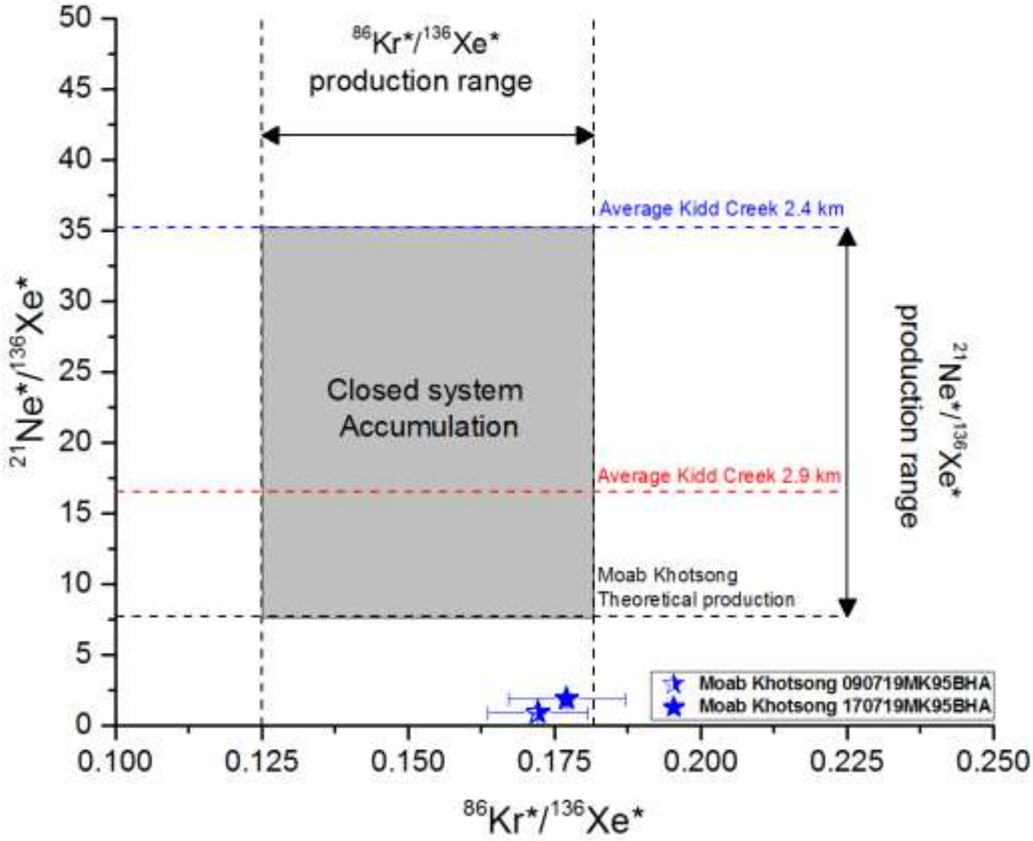

As with Fig. 3, the ^{86}Kr*/^{136}Xe* production range (between the vertical black hatched lines) is taken from Ballentine and Burnard[30]. The theoretical ^{21}Ne*/^{136}Xe* production rate for Moab Khotsong (horizontal black hatched line) is calculated based on the theoretical ^{4}He*/^{136}Xe*from uranium decay coupled with the site-specific ^{4}He*/^{21}Ne* production ratio of 3.95×10^7 (Methods). For additional context, as with Fig. 3, average ^{21}Ne*/^{136}Xe* production ranges are plotted for fracture fluids from two depths of Kidd Creek, Canada (blue and red hatched lines), which is considered representative of a closed system given the good agreement among ^{4}He*, ^{21}Ne*, ^{40}Ar*, and ^{136}Xe*-derived residence times[2,14]. Both Kidd Creek lines are based on a ^{4}He*/^{21}Ne* production ratio of 9.96×10^6 as per published studies[2,14,30]. The shaded grey box then suggests the space that defines a closed system and incorporates ^{21}Ne production in both a high and low uranium environment. As with ^{4}He* (Fig. 3), both Moab Khotsong Mine brines (blue stars) reveal significant depletion in ^{21}Ne*/^{136}Xe* with respect to closed system accumulation, but retain ^{86}Kr*/^{136}Xe* ratios consistent with in situ fissiogenic production from uranium. As atmospheric addition serves to reduce radiogenic excesses proportionally, both samples preserve in situ radiogenic ratios. Such a pattern of depletion in the light noble gases ^{4}He (and ^{21}Ne) with respect to their heavy noble gas counterparts is most consistent with diffusive loss. Error bars represent full propagated analytical uncertainty of 1σ.

Supplementary Table 1: Measured gas phase noble gas concentrations in cm^3/cm^3 of sample at STP and water isotope data

Sample	^{3}He	±	^{4}He	±	^{20}Ne	±	^{21}Ne	±	^{22}Ne	±	^{36}Ar	±
**090719MK95BHA**	3.0E-09	1.2E-10	1.0E-01	3.3E-03	2.3E-07	6.0E-10	4.5E-09	1.2E-11	2.5E-08	6.6E-11	7.3E-07	2.3E-08
**170719MK95BHA**	2.9E-09	9.6E-11	1.0E-01	2.9E-03	6.8E-08	1.7E-10	3.8E-09	9.5E-12	8.6E-09	2.2E-11	3.3E-07	1.6E-08
**Air**	7.3E-12	1.1E-13	5.2E-06	5.0E-08	1.6E-05	1.4E-07	4.9E-08	5.2E-10	1.7E-06	3.7E-09	3.1E-05	3.5E-08
**ASW (20°C Seawater)**	5.2E-14	7.8E-16	3.7E-08	3.6E-10	1.4E-07	1.2E-09	4.1E-10	4.3E-12	1.4E-08	3.1E-11	8.3E-07	9.3E-10

Sample	^{38}Ar	±	^{40}Ar	±	^{78}Kr	±	^{80}Kr	±	^{82}Kr	±	^{83}Kr	±
**090719MK95BHA**	1.3E-07	7.4E-09	1.5E-02	2.9E-05	1.3E-10	4.2E-12	8.6E-10	1.7E-11	4.4E-09	1.4E-10	4.4E-09	1.3E-10
**170719MK95BHA**	6.2E-08	5.6E-09	1.6E-02	2.6E-05	5.4E-11	2.1E-12	3.5E-10	8.2E-12	1.8E-09	6.8E-11	1.8E-09	6.7E-11
**Air**	5.9E-06	1.5E-08	9.3E-03	1.9E-05	4.0E-09	4.1E-11	2.6E-08	2.5E-10	1.3E-07	1.3E-09	1.3E-07	1.3E-09
**ASW (20°C Seawater)**	1.6E-07	3.9E-10	2.5E-04	5.0E-07	1.9E-10	2.0E-12	1.2E-09	1.2E-11	6.3E-09	6.1E-11	6.3E-09	6.3E-11

Sample	^{84}Kr	±	^{86}Kr	±	^{124}Xe	±	^{126}Xe	±	^{128}Xe	±	^{129}Xe	±
**090719MK95BHA**	2.2E-08	6.7E-10	7.0E-09	2.0E-10	5.2E-12	1.3E-13	4.8E-12	1.2E-13	1.0E-10	2.6E-12	1.4E-09	3.6E-11
**170719MK95BHA**	8.8E-09	3.3E-10	2.9E-09	1.0E-10	3.1E-12	8.5E-14	2.9E-12	7.9E-14	6.2E-11	1.7E-12	8.4E-10	2.3E-11
**Air**	6.5E-07	6.3E-09	2.0E-07	2.0E-09	7.7E-11	1.2E-12	7.2E-11	1.2E-12	1.6E-09	2.4E-11	2.1E-08	3.2E-10
**ASW (20°C Seawater)**	3.1E-08	3.0E-10	9.5E-09	9.5E-11	8.8E-12	1.4E-13	8.2E-12	1.4E-13	1.8E-10	2.8E-12	2.5E-09	3.7E-11

Sample	^{130}Xe	±	^{131}Xe	±	^{132}Xe	±	^{134}Xe	±	^{136}Xe	±	$\delta^{18}O$	$\delta^{2}H$
**090719MK95BHA**	2.2E-10	4.7E-12	1.3E-09	3.3E-11	2.3E-09	3.6E-11	1.8E-09	4.8E-11	2.0E-09	5.3E-11	-12.2	-25.4
**170719MK95BHA**	1.3E-10	3.0E-12	7.9E-10	2.2E-11	1.6E-09	2.7E-11	1.4E-09	4.1E-11	1.6E-09	4.6E-11	-12.3	-25.8
**Air**	3.3E-09	5.2E-11	1.7E-08	2.7E-10	2.2E-08	3.4E-10	8.4E-09	1.3E-10	7.2E-09	1.1E-10	N/A	N/A
**ASW (20°C Seawater)**	3.8E-10	5.9E-12	2.0E-09	3.1E-11	2.5E-09	3.9E-11	9.7E-10	1.5E-11	8.2E-10	1.2E-11	-4.0[a]	-23.9[a]

All values have been corrected for full procedural blanks. Reference air and ASW concentrations are taken from published sources[31,32]. Corresponding water isotope data ($\delta^{18}O$ and $\delta^{2}H$) for these samples in per mil (‰) are also taken from the literature and reveals this fracture network has been hydrogeologically isolated from local meteoric water sources over significant geological time[9]. [a]Published water geochemical data from an overlying aquifer which lies on the Global Meteoric Water line (GMWL) is here considered representative of the local meteoric water source[9]. Error bars for noble gas data represent full analytical uncertainty at 1σ. Published error bars for $\delta^{18}O$ and $\delta^{2}H$ are 0.2‰ and 0.8‰ respectively.

Supplementary Table 2: Measured gas phase noble gas ratios

Sample	^{3}He/^{4}He (R$_{A}$)	±	^{20}Ne/^{22}Ne	±	^{21}Ne/^{22}Ne	±	^{38}Ar/^{36}Ar	±	^{40}Ar/^{36}Ar	±	^{78}Kr/^{80}Kr	±
**090719MK95BHA**	0.0210	0.0004	9.079	0.002	0.17896	0.00006	0.175	0.009	20625	635	0.1532	0.0038
**170719MK95BHA**	0.0199	0.0003	7.848	0.003	0.43753	0.00011	0.188	0.014	48617	2378	0.1540	0.0047
Kidd Creek 2.4km[14]	*0.014*	*0.002*	*7.6*	*0.4*	*0.34*	*0.05*	*0.1875*	*0.0003*	*31138*	*7887*	*N/A*	*N/A*
Kidd Creek 2.9km[2]	*0.0256*	*0.0006*	*5.3*	*0.2*	*0.60*	*0.02*	*0.209*	*0.022*	*102365*	*22012*	*0.16013*	*0.0004*
Mt Isa[34]	*N/A*	*N/A*	*9.0*	*0.4*	*0.09*	*0.07*	*N/A*	*N/A*	*7904*	*10513*	*N/A*	*N/A*
**Air/ASW**	1.00	0.01	9.80	0.08	0.0290	0.0003	0.1880	0.0006	295.5	0.7	0.154	0.001

Sample	^{82}Kr/^{80}Kr	±	^{83}Kr/^{80}Kr	±	^{84}Kr/^{80}Kr	±	^{86}Kr/^{80}Kr	±	^{124}Xe/^{130}Xe	±	^{126}Xe/^{130}Xe	±
**090719MK95BHA**	5.1	0.1	5.1	0.1	25.1	0.6	8.1	0.2	0.0238	0.0003	0.0221	0.0003
**170719MK95BHA**	5.0	0.2	5.1	0.1	25.0	0.8	8.3	0.2	0.0239	0.0003	0.0222	0.0003
Kidd Creek 2.4km[14]	*N/A*	*N/A*	*N/A*	*N/A*	*N/A*	*N/A*	*N/A*	*N/A*	*0.0238*	*0.0001*	*0.0221*	*0.0001*
Kidd Creek 2.9km[2]	*5.07*	*0.02*	*5.07*	*0.02*	*25.1*	*0.1*	*8.0*	*0.1*	*0.0241*	*0.0001*	*0.0221*	*0.0002*
Mt Isa[34]	*N/A*	*N/A*	*N/A*	*N/A*	*N/A*	*N/A*	*N/A*	*N/A*	*N/A*	*N/A*	*N/A*	*N/A*
**Air/ASW**	5.11	0.03	5.08	0.03	25.3	0.2	7.71	0.05	0.0234	0.0003	0.0218	0.0003

Sample	^{128}Xe/^{130}Xe	±	^{129}Xe/^{130}Xe	±	^{131}Xe/^{130}Xe	±	^{132}Xe/^{130}Xe	±	^{134}Xe/^{130}Xe	±	^{136}Xe/^{130}Xe	±
**090719MK95BHA**	0.474	0.007	6.48	0.09	5.90	0.08	10.5	0.2	8.2	0.1	9.1	0.1
**170719MK95BHA**	0.477	0.007	6.51	0.09	6.12	0.09	12.4	0.2	11.0	0.2	12.4	0.2
Kidd Creek 2.4km[14]	*0.4751*	*0.0007*	*6.572*	*0.006*	*5.242*	*0.008*	*6.83*	*0.03*	*2.94*	*0.04*	*2.64*	*0.05*
Kidd Creek 2.9km[2]	*0.482*	*0.003*	*6.75*	*0.04*	*5.5*	*0.3*	*7.58*	*0.07*	*4.13*	*0.09*	*4.1*	*0.1*
Mt Isa[34]	*0.48*	*0.02*	*6.6*	*0.6*	*5.8*	*1.0*	*7.2*	*0.9*	*3.5*	*1.1*	*3.35*	*1.48*
**Air/ASW**	0.472	0.006	6.50	0.08	5.21	0.07	6.61	0.09	2.56	0.03	2.18	0.03

All values for Moab Khotsong represent full procedural analytical uncertainty. Published air/ASW ratios[31] are presented in bold and the associated uncertainties here incorporate both propagated uncertainty in the measured air standards and the published uncertainty. For additional context, average and standard deviation ratios for previously reported free flowing fracture fluid ratios for Kidd Creek 2.4 km[14], Kidd Creek 2.9 km[2] and fluid inclusion data from Mt Isa[34] are also presented (grey italics). In the Kidd Creek 2.9 km fracture fluids a modest deviation in ^{78}Kr/^{80}Kr and ^{86}/Kr/^{80}Kr was noted by Warr et al., at concentrations an order of magnitude lower than those presented here, However, these did not coincide with neither any mass fractionation or residence time trends and were not considered significant[2]. N/A indicates data not available.

Supplementary Table 3: Reconstructed concentrations of radiogenic excesses in cm³/cm³ of fluid at STP

Sample	$^{4}He^{*}$	±	$^{21}Ne^{*}$	±	$^{40}Ar^{*}$	±	$^{86}Kr^{*}$	±	$^{136}Xe^{*}$	±
**090719MK95BHA**	6.22E-02	1.97E-03	2.30E-09	8.62E-12	1.69E-02	5.22E-04	4.47E-10	1.57E-11	2.61E-09	9.04E-11
**170719MK95BHA**	2.12E-01	5.95E-03	7.26E-09	2.57E-11	4.02E-02	1.97E-03	6.79E-10	3E-11	3.9E-09	1.44E-10

The process to calculate initial fluid concentrations is outlined in the methods section. Error bars represent full analytical uncertainty at 1σ.

Supplementary Table 4: Measured gas phase concentrations in vol % at STP for a previous sample collected from the same locality

Sample	He	Ar	H_2	O_2	N_2	CH_4	C_2H_6	C_3H_8	i-C_4H_{10}	nC_4H_{10}	i-C_5H_{12}	n-C_5H_{12}	Total
**231018MK95BHA**	8.23	1.36*	4.83	0*	3.38*	76.73	5.06	0.41	<0.05	<0.05	<0.05	<0.05	100

Stated analytical uncertainty is 5% as with previous analyses[4,6,46,54]. Sample has been air corrected for components with the * symbol and normalized. He and Ar concentrations reveal comparable concentrations to the noble gas isotope data (Supplementary Table 1), providing independent validation of the dataset at 1σ.

Supplementary Table 5: Noble gas apparent residence times in Ga

Sample	^{4}He (Ga)	^{21}Ne (Ga)	^{40}Ar (Ga)	^{136}Xe (Ga)
**090719MK95BHA**	*0.32 ± 0.14*	*0.46 ± 0.21*	0.60 ± 0.27	*3.18 ± 1.44*
**170719MK95BHA**	*1.03 ±0.47*	*1.36 ±0.62*	1.20 ± 0.54	*4.11 ± 1.87*

Residence times are derived by incorporating the radiogenic excesses (Supplementary Table 2) into the approach outlined fully in the methods using a host rock radioelement concentration of 2.33 ppm (U) 10.9 ppm (Th), and 1.91% (K)[10,35]. Sample 090719MK95BHA reveals notably lower residence times for all samples, in line with trace atmospheric addition[14], and so residence times from this sample are not considered representative of the system. For sample 170719MK95BHA, which is considered representative of the system, given the identified additional influence of the U-rich Vaal Reef on ^{4}He, ^{21}Ne, and ^{136}Xe, coupled with the diffusive loss of He and Ne, only the ^{40}Ar residence times are considered representative, as these residence time estimates are only dependent on K concentration – See main text[30]. Residence times listed in grey italics denote samples affected by air contamination and/or reef inputs and therefore do not accurately represent fluid residence times for Moab Khotsong. Uncertainty incorporates propagated porosity error, analytical error, and variability in U, Th, K concentrations following the methods of Warr et al.[2]. As with previous studies the variability in porosity estimates represents the dominant source of uncertainty[2,3,14,17].
